# Water Adsorption Dynamics on Metal–Organic Framework MOF-801: Comparative Study of Loose and Glued Grains, and Coatings

**DOI:** 10.3390/nano13172442

**Published:** 2023-08-28

**Authors:** Marina Solovyeva, Larisa Gordeeva

**Affiliations:** Boreskov Institute of Catalysis, Ak. Lavrentiev av. 5, 630090 Novosibirsk, Russia; solovyeva@catalysis.ru

**Keywords:** adsorption heat transformation and storage, MOF-801, water adsorption dynamics, compact adsorbent beds, binder chemical nature, specific power

## Abstract

Adsorption heat transformation and storage (AHTS) is an environmentally benign and energy-saving alternative to common compression chillers and heat pumps. The low specific power (SP) of adsorption systems is a key drawback that hinders their broader dissemination. The optimization of adsorption dynamics is a prerequisite for SP enhancement. In this work, we studied the dynamics of water vapor adsorption on MOF-801—a promising adsorbent for AHTS. For the first time, two configurations of compact MOF-801 bed, namely, grains glued to the surface of a metal support and thin adsorbent coatings, are prepared, and their porous structure is characterized. The water adsorption dynamics is compared with a common loose grains configuration. The main findings are: (a) the binder can both accelerate and decelerate the water adsorption, and its chemical nature is subject to careful selection; (b) at the initial time, heat transfer between the support and adsorbent bed controls the adsorption rate, and, at a longer time, mass transfer starts to affect it; (c) polyvinylpyrrolidone, bentonite, thermal grease, and hydroxyethylcellulose increase the heat transfer coefficients and accelerate adsorption; polyvinyl alcohol and polyaniline slow it down; and (d) for the coatings prepared with polyvinylpyrrolidone, an SP of 1.6–5.1 kW/kg can be achieved, which is about twice that for the loose grains. The new configurations can be recommended for effective but expensive adsorbents such as MOFs, and their implementation will contribute to the further spread of AHTS technology.

## 1. Introduction

According to the British Petroleum (BP) statistical review, the increase in primary energy consumption was equal to 31 × 10^18^ J in 2021, which was above the pre-pandemic level in 2019, with an associated growth of CO_2_ emissions of 5.9% [1]. For this reason, global energy policies in recent years have been focused on the importance of decarbonizing energy. Adsorption heat transformation and storage (AHTS) is an emerging technology, which enables efficient use of renewable or waste heat sources for cooling/heating [2]. Due to the low consumption of electric power and the use of green working fluids (water, methanol, and ammonia), AHTS is considered an energy-saving and environmentally friendly alternative to common compression chillers and heat pumps. It is expected that the widespread of AHTS can lead to a significant reduction in fossil fuel consumption and greenhouse gas emissions. Nevertheless, a broader diffusion of this technology is limited by the low specific power (SP) achievable in practice, and the associated large size of AHTS units [3].

The SP of AHTS systems is mainly affected by the dynamics of the adsorption and desorption stages of the working cycle, governed by coupled heat and mass transfer in the adsorber–heat exchanger (Ad-HEx) unit. In this vein, the optimization of the Ad-HEx configuration is becoming a hot topic of research related to AHTS technology [4,5,6]. Nowadays, two basic Ad-HEx configurations are widely used, namely, the loose adsorbent grains loaded between the HEx fins and the thin adsorbent coating on the HEx surface [7,8]. Due to the large porosity of the loose grains configuration and high contact resistance, the poor heat transfer between the adsorbent bed and HEx surface and inside the bed is considered to be the factor controlling the adsorption rate and reducing the SP [9,10,11]. An effective way to improve heat transfer between the adsorbent and HEx is to apply the adsorbent coating on the HEx surface with or without a binder [12,13,14]. In this case, the point contact between the adsorbent grains and the HEx surface is substituted by the tight contact between the adsorbent coating and the HEx surface; therefore, the coatings provide a strong reduction in the contact resistance and an increase in the heat transfer coefficient [15]. However, the small porosity of the coatings leads to the mass transfer in the adsorbent bed becoming slower, which can become a new factor, limiting the adsorption kinetics and the SP of the coated Ad-HEx [16,17]. Consequently, a smart trade-off between heat and mass transfer is needed to accelerate the ad/desorption and improve the SP. This compromise is complicated by the fact that both heat and mass transfer are inevitably coupled in the Ad-HEx because a large amount of heat is released/consumed during vapor ad/desorption [3,18]. Meanwhile, the correct and accurate comparison of adsorption dynamics for the loose grains and coated HEx configurations is often challenging due to different grain sizes, adsorbent masses, bed thickness, contact surface area, etc. All these factors affect both heat and mass transfer, which makes it difficult to reveal the effect of consolidation of the adsorbent with the HEx surface on the adsorption dynamics. Accordingly, there is room for optimizing the Ad-HEx configuration. In particular, the influence of the binder nature and content on the kinetics of adsorption on various adsorbents has not been sufficiently studied.

A class of porous compounds known as metal–organic frameworks (MOFs) have attracted increasing attention in recent years as adsorbents for AHTS due to their high porosity, huge surface area, and tunable adsorption properties [19,20]. MOF-801 is a microporous crystalline compound, which consists of basic units of Zr_6_O_4_(OH)_4_(–O_2_C–CH=CH–CO_2_–)_6_ and forms a cubic structure with two tetrahedral cages, with sizes of 4.8 and 5.6 Å, and an octahedral cage, with size of 7.4 Å. MOF-801 is characterized by a high water adsorption capacity of 0.36 g/g at relative pressure *P/P*_0_ = 0.9 [21]. Owing to its microporous structure and a high crystal density of 1.68 g/cm^3^, the adsorption capacity related to the unit volume reaches 0.60 g/cm^3^ [21]. Due to the peculiar stepped shape of the characteristic curve of water adsorption (Figure S1a in the Supplementary Materials) and relatively high hydrophilicity, MOF-801 enables a chilling effect at a low evaporation temperature of 5 °C and adsorption temperature of 30 °C, can be regenerated at a low temperature of 80–85 °C, and exchanges 0.21–0.23 g_water/g_ads per cycle [22]. Furthermore, the structure and properties of MOF-801 can be tuned by the linker functionalization [23] and partial substitution of Zr by other metals (Co and Ni) [24]. Recently, several synthetic approaches have been developed using water instead of toxic organic solvents at room temperature, which enables simple, energy-efficient, and environmentally friendly production of MOF-801 [25,26]. These features, along with its exceptional hydrothermal stability, make MOF-801 one of the most promising water adsorbents for energy conversion [22,25], climate control [27], and atmospheric-water-harvesting systems [28,29]. However, the microcrystalline powder form of as-synthesized MOFs limits their practical application. Accordingly, shaping MOFs in the form of pellets or coatings suitable for handling, processing, and storage is one of the strategies that contribute to their scale-up application. The method of compression of neat MOF-801 or MOF-801 mixed with binders (polyvinyl alcohol, sucrose, and polyvinyl butyral) was used in [30] to produce pellets. Gökpinar et al. described the freeze granulation method using polyvinyl alcohol as a binder, which allows MOF-801 pellets to be produced with essentially unchanged porosity [31]. He et al. [32] constructed monolithic MOF-801 by sol–gel phase separation followed by acid–heat post-treatment. Monolithic MOF-801 showed a moisture sorption capacity 1.2–1.4 times higher than that of conventional powdered and compressed samples.

**Figure 1:**
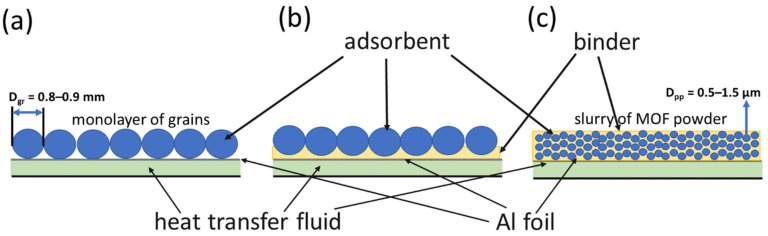
The studied adsorbent bed configurations: common—loose grains (**a**), adsorbent/binder coating (**c**), and innovative—a consolidated layer of glued grains on the heat exchanger (HEx) surface (**b**).

This work presents the results of the comparative study of the water sorption dynamics on three usable configurations of MOF-801 bed under conditions of a typical cooling cycle. The first configuration is a monolayer of loose MOF-801 grains placed on the surface of aluminum foil, which imitates the surface of aluminum HEx, often used in AHTS systems (Figure 1a). The second is an innovative configuration, composed of a monolayer of MOF-801 grains glued to the foil surface with a binder (Figure 1b). The last is the coating, prepared from the slurry of MOF-801 powder in the aqueous solution of binders using a drop-coating method (Figure 1c). We focused on the effects of the binder’s chemical nature and content and the coating thickness on the adsorption kinetics. To reveal the effect of the adsorbent layer consolidation with the support, we fixed other factors influencing the kinetics, namely, the adsorbent mass, the contact surface area between the adsorbent and the foil, and the adsorbent grain size (for loose and glued grains). Based on the obtained results, the rate-limiting stages are revealed for each configuration, the effective heat transfer coefficient, α, between the aluminum support and the adsorbent bed is evaluated under conditions of the cooling cycle, and the SP achievable employing the studied adsorbent bed configurations is estimated. Appropriate recommendations are proposed for optimizing the adsorbent bed configuration to increase the SP of AHTS.

## 2. Materials and Methods

### 2.1. Sample Synthesis

MOF-801 was synthesized by a solvothermal method according to a slightly modified procedure described in [21]. ZrOCl_2_·8·H_2_O (3.2 g, 10 mmol) and fumaric acid (1.2 g, 10 mmol) were dissolved in a solution of dimethylformamide (DMF)/formic acid (40 mL/14 mL), and then the mixture was placed into a 100 mL Teflon-lined autoclave and heated at 130 °C for 6 h. After cooling to room temperature, the mixture was filtered and washed with DMF and methanol. Then, the white solid precipitate obtained was dried at 150 °C for 24 h at continuous evacuation, yielding an activated sample of MOF-801.

To prepare MOF-801 grains, the synthesized MOF-801 powder was pressed, milled, and sieved into fractions, with the grain sizes D_gr_ = 0.4–0.5 and 0.8–0.9 mm (Figure S2 in the Supplementary Materials). Consolidated layers of MOF-801 were prepared with organic (polyvinyl alcohol (PVA), polyvinylpyrrolidone (PVP), and polyaniline (PAN)), inorganic (bentonite, aluminum oxynitrate (AON), and heat-conductive compound CPTD), and hybrid (thermal grease Aerocool Cog (China)) binders. To prepare the glued grains configuration (Figure 1b), the appropriate amount of the binder was added to distilled water and mixed on a magnetic stirrer. A disc of fixed diameter (1.2 and 1.7 mm for monolayers of 0.8–0.9 and 0.4–0.5 mm grains, respectively) made of Al foil was treated with acetone for degreasing, and the obtained binder–water solution/slurry was applied to it as a thin layer and weighted. Then, MOF-801 grains with a mass *m*_MOF-801_ = 100 mg were evenly distributed on to the disc to form a monolayer, and the foil with the binder and MOF-801 was weighed again. The minimum binder content was chosen, sufficient for preparing a strong layer. The strength of the compact layers was estimated by visual control; the layers were not destroyed or exfoliated under touch stress and after numerous (ca. 10) adsorption–desorption cycles. To synthesize the MOF-801 coating (Figure 1b), the slurry consisting of the known amount of MOF-801 powder, binder, and water was applied to pretreated Al foil using the drop-coating method (Figure S2 in the Supplementary Materials) [7]. The total mass of the slurry deposited on the foil was calculated as

*m*_sl_ = *m*_MOF-801_/*C*_MOF-801_, (1)

where *C*_MOF-801_ is the mass fraction of MOF-801 in the slurry. Thus, the MOF-801 mass was the same (*m*_MOF-801_ = 100 ± 2 mg) for all the studied configurations. The prepared MOF-801 wet beds were dried at room temperature for 3 h and then at 100 °C for 16 h. The binder content and the coating thickness were varied.

### 2.2. Adsorbent Characterization

The structure of prepared MOF-801 was confirmed by powder X-ray diffraction (PXRD) using a Bruker D8 diffractometer with an XRK-900 reactor chamber. The diffraction patterns were recorded using CuK_α_ radiation in the range of 2θ from 5° to 60°. Measurements were carried out in the scan mode with 0.02° increments and an accumulation time of 10 s at each point. The porous structures of the synthesized MOF-801 and the prepared compact layers were studied using low-temperature N_2_ adsorption at 77 K on a Quantachrome Nova 1200 gas sorption analyzer. The specific surface area, *S*_sp_, was calculated using BET analysis of the adsorption branch of the isotherm in the relative pressure range of 0.01–0.07. The total pore volume, *V_p_*, was calculated from the amount of N_2_ adsorbed at a relative pressure *P/P*_0_ = 0.99. The micropore volume, *V*_μ_, was calculated using the statistical thickness analysis of the isotherm adsorption branch and de Boer’s t-method. The morphology of the prepared beds was characterized with a Stemi 2000-C optical microscope and a scanning electron microscope Regulus 8230 (Hitachi, Tokyo, Japan) with cold field emission at probe electron energies of 2.5 and 4 kV.

### 2.3. Water Adsorption Dynamics

The water adsorption dynamics on MOF-801 were studied using a large temperature jump method [33] under operating conditions of isobaric stages of a typical adsorptive cooling cycle (the evaporation, condensation, adsorption, and desorption temperatures *T*_ev_/*T*_con_/*T*_ads_/*T*_des_ = 5/30/30/80 °C, respectively). The experimental rig, consisting of a measuring cell, a buffering vessel, and an evaporator filled with distilled water, is described in more detail elsewhere [22,33].

The beds MOF-801-G_*S*/*m* composed of monolayers of loose MOF-801 grains (mass *m*_MOF-801_ = 100 ± 2 mg), were deposited on Al foil of 100 μm thickness and placed on the bottom of the measuring cell. To reduce the contact resistance between the Al foil and the measuring cell bottom, the foil was glued to the bottom with heat-conductive compound CPTD. The effect of Al foil on the water adsorption kinetics is minor (Figure S3 in the Supplementary Materials). Two fractions of loose grains of 0.4–0.5 and 0.8–0.9 mm sizes were used, which correspond to the contact surface, *S*, between the monolayer of grains and the foil of 8.9 and 4.5 cm^2^ (or the ratio *S*/*m* = 8.9 and 4.5 m^2^/kg), respectively. The beds MOF-801-G/binder_*S*/*m* of glued grains were prepared using 0.8–0.9 mm grains. When preparing coatings MOF-801-C/binder (C_b_,wt%)_*S*/*m*, we fixed the MOF-801 mass *m*_MOF-801_ = 100 ± 2 mg and the ratio *S*/*m* = 8.9 and 4.5 ± 0.1 m^2^/kg, which correspond to the reference beds of loose grains of 0.4–0.5 and 0.8–0.9 mm sizes, respectively. This allows a thorough comparison of the sorption dynamics for grains and coatings and reveals the effects of the bed configuration and binder nature.

The adsorption and desorption kinetics were measured at the vapor pressures *P*_ev_ = 9 mbar and *P*_con_ = 43 mbar, which correspond to the temperatures of the evaporator *T*_ev_ = 5 °C and condenser *T*_con_ = 30 °C, respectively. During adsorption runs, the temperature of the metal support was dropped from the initial temperature *T*_ads.in_ = 60 °C to the final temperature *T*_ads_ = 30 °C. For desorption runs, the temperature was increased from *T*_des.in_ = 55 °C to *T*_des_ = 80 °C. The initial temperatures of adsorption and desorption were selected according to the isosteric chart of the “MOF-801–water” pair (Figure S1b in the Supplementary Materials) [22]. The water vapor ad/desorption caused the decrease/increase in the pressure over the adsorbent, which did not exceed 1.2 mbar; thus, quasi-isobaric conditions typical of the adsorption cooling cycle were maintained. The amount of water ad/desorbed, *m*_H2O_, was calculated by using the ideal gas equation from the evolution of vapor pressure, *P*(*t*). The water uptake, Δ*w*(*t*) (g/g), was related to the unit mass of MOF-801 and calculated as

Δ*w*(*t*) = *m*_H2O_(*t*)/*m*_MOF-801_ = *m*_H2O_(*t*), (2)

where *m*_MOF-801_ is the mass of MOF-801 loaded to the measuring cell. The dimensionless conversion was calculated as

*q* = Δ*w*(*t*)/Δ*w_t_*_→∝_, (3)

where Δ*w_t__→∝_* is the final uptake variation.

## 3. Results and Discussion

### 3.1. Characterization of the MOF-801 Consolidated Beds

**Figure 2:**
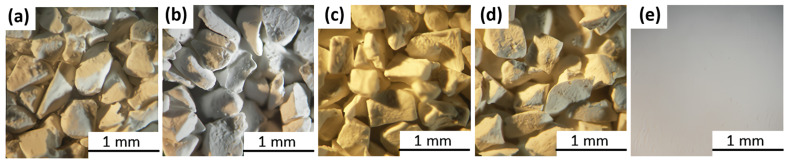
Optical microscopic images of loose grains MOF-801_G_4.5 (**a**), glued grains MOF-801_G/CPTD_4.5 (**b**), MOF-801_G/PVP_4.5 (**c**), MOF-801_G/bentonite_4.5 (**d**), and coating MOF-801_C/PVP(10)_8.9 (**e**).

**Figure 3:**
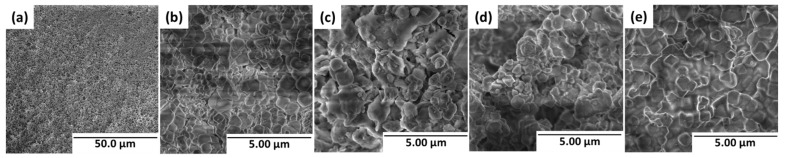
SEM images of loose grains MOF-801_G_4.5 (**a**,**b**), glued grains MOF-801_G/bentonite_4.5 (**c**), MOF-801_G/PVP_4.5 (**d**), and coating MOF-801_C/PVP(10)_8.9 (**e**).

The optical microscope images show that the morphology of the MOF-801 grains glued to Al foil remains intact (Figure 2a–d). The binder particles are not observed on the grains’ surface. The coating is homogeneous, and a slight roughness is detected on its surface (Figure 2e). SEM images of MOF-801 show that loose MOF-801 grains are constituted by an assemblage of primary particles of irregular shape and 0.5–1.5 μm in size (Figure 3a,b). These primary particles are observed also in SEM images of glued grains and coatings. The shape and size of the primary particles are similar for loose and glued grains and coatings. A slight smoothening can be detected in the coating (Figure 3e), probably due to the binder (PVP).

According to low-temperature N_2_ adsorption data (Figure S4a and Table 1), as-prepared MOF-801 powder is meso-microporous, with a high specific surface area of 900 m^2^/g and total pore volume of 0.49 cm^3^/g (Table 1). The experimental PXRD pattern (Figure S4b) is consistent with the literature data and the pattern simulated from single-crystal X-ray data [21]. These results confirm the genuine structure and high porosity of the synthesized MOF-801.

Pressing powder to form grains leads to a slight decrease in the specific surface area to 820 m^2^/g and the pore volume to 0.45 cm^3^/g. Using the binders further changes the texture characteristics of the compact beds. The pore size distribution, calculated using the Dubinin–Astakhov model, is similar for loose grains and compact layers (Figure S5 in the Supplementary Materials).

To characterize the influence of the binder on the texture characteristics of MOF-801, the clogging degree is calculated as

*δ =* (*V*_p_
*− V*_p_l_)*/V*_p_ × 100%, (4)

where *V*_p_ and *V*_p_l_ are the specific pore volumes of the pristine MOF-801 and consolidated layers, respectively. When calculating *δ* for the glued grains and coatings, *V*_p_ was taken for MOF-801 grains and the powder, respectively.

The clogging degree is minor (*δ* = 0–11%) for organic binders PVA, PVP, PAN, and HEC, and thermal grease Aerocool (Table 1). CPTD strongly reduces the specific pore volume due to partial clogging of the pores of MOF-801, or destroys its structure, with the degree *δ* = 29%. On the contrary, using bentonite as a binder increases both specific pore volume and surface area; the degree, δ, formally calculated according to Equation (2), is negative for the compact layers prepared with bentonite (Table 1). A probable reason could be the intrinsic porosity of bentonite clay. Bentonite is a layered clay mineral with interlayer and intergranular porosity and a total pore volume of about 0.1–0.2 cm^3^/g [34,35]. Accordingly, the addition of porous bentonite to MOF-801 increases the specific pore volume and surface area of the layers related to the unit mass of the MOF.

### 3.2. Water Vapor Adsorption Dynamics

#### 3.2.1. Glued MOF-801 Grains

First, the final uptake variation, Δ*w*_t→∝_ = 0.22 ± 0.01 g/g, was measured for loose grains of MOF-801 under conditions of the cooling cycle (*T*_ev_/*T*_ads_/*T*_des_ = 5/30/80 °C, respectively) (Table 2). It is close to the uptake variation, Δ*w* = 0.21 g/g, calculated for the considered cycle from the isosteric chart of water adsorption for as-synthesized MOF-801 powder (Figure S1b in the Supplementary Materials) [22]. Most binders used for gluing the grains to Al foil change the uptake variation related to the unit mass of MOF-801 only slightly: Δ*w*_t→∝_ = 0.20–0.24 g/g for MOF-801_G/binder_4.5 beds. Thus, they have little effect on the adsorption capacity of MOF-801. Aluminum oxynitrate reduces dramatically the water uptake on glued MOF-801 grains to Δ*w*_t→∝_ = 0.12 ± 0.01 g/g. The degree δ = 9% for MOF-801/AON is moderate; consequently, pore blockage is unlikely to cause such a decrease in uptake variation. Probable reasons for the reduction might be: (i) a chemical interaction of AON with the adsorption sites of MOF-801, leading to their deactivation; or (ii) a change in the crystallinity of MOF-801, which also has a strong effect on its adsorption properties [21]. This sample was no longer considered.

**Figure 4:**
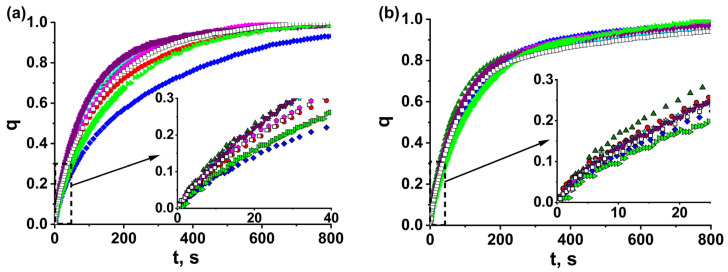
The kinetic curves of water vapor adsorption (**a**) and desorption (**b**) for glued MOF-801 grains MOF-801_G/CPTD_4.5 (,), MOF-801_G/Aerocool_4.5 (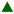), MOF-801_G/PVA_4.5 (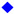), MOF-801_G/PVP_4.5 (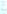), MOF-801_G/bent_4.5 (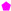), MOF-801_G/HEC_4.5 (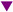), MOF-801_G/PAN_4.5 (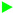), and the reference bed of loose grains MOF-801_G_4.5 (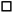). D_gr_ = 0.8–0.9 mm.

The kinetic curves of water adsorption and desorption “dimensionless uptake, *q*, vs. time, *t*,” for glued grains MOF-801_G/binder_4.5 and reference loose grains MOF-801_G_4.5 of 0.8–0.9 mm size (Figure 4) are monotonically increasing lines approaching *q* = 1. When presented in coordinates of the first-order equation “ln(1 *− q*) vs. *t*”, the kinetic curves turn into two straight lines (Figures S6 and S7 in the Supplementary Materials), indicating that they can be described by the sum of two exponents

*q*(*t*) *= A* [1 *−* exp(*−t/τ*_1_)] + (1 *− A*) [1 *−* exp(*−t/τ*_2_)], (5)

where τ_1_ and τ_2_ are the characteristic times and A is the fitting parameter (Table 2). This may show that, during ad/desorption, the rate-limiting process changes. The characteristic time *τ*_1_ = 67–130 and 91–145 s for adsorption and desorption runs, respectively, describes the initial fast process. The second time, τ_2_ = 130–330 and 270–460 s, respectively, is longer for both runs and is associated with a further slower process. The relative error in determining the characteristic times is 10%. The coefficient A equal to (0.11–0.29) ± 0.05 for adsorption shows the fraction, *q*, of water adsorbed according to the first fast process. For desorption runs, the transition to the slow process occurs at a much higher conversion: *q* = A = (0.53–0.69) ± 0.05.

For reference bed MOF-801-G_4.5 of loose grains, the characteristic times are τ_1_ = 88 and 120 s and τ_2_ = 170 and 480 s for adsorption and desorption, respectively, (Table 2). Gluing the adsorbent grains to Al foil by binders can lead to both acceleration and deceleration of water ad/desorption (Figure 3). For the grains glued by Aerocool, bentonite, PVP, and HEC, the characteristic time, *τ*_1_, which describes the initial fast process, decreases to 67–77 s. A similar effect is observed for desorption, where time *τ*_1_ diminishes to 91–106 s. On the contrary, PVA and PAN essentially decelerate ad/desorption, and time *τ*_1_ rises (Table 2). This is quite surprising, since the grains are glued to Al foil with binder, and the contact surface area between the grains and the foil increases, thus decreasing the contact resistance to the heat transfer. Accordingly, an increase in the sorption rate can be expected.

In actual practice, the duration of ad/desorption stages is usually limited by the conversion *q* = 0.7–0.8 to avoid slowing down the sorption when approaching equilibrium (*q* = 1). Accordingly, for the convenience of comparing different adsorbent bed configurations, the times *t*_0.7_ and *t*_0.8_, corresponding to the conversion *q* = 0.7 and 0.8, respectively, were determined from the sorption kinetics (Table 2). Similar to time *τ*_1_, a downward trend was also observed for times t_0.7_ and t_0.8_ for the grains glued by Aerocool, bentonite, PVP, and HEC (Table 2).

Thus, the effect of gluing the MOF-801 grains to Al foil on the water sorption kinetics strongly depends on the binder’s chemical nature. Aerocool, bentonite, PVP, and HEC accelerate ad/desorption. On the contrary, PVA and PAN slow down them.

#### 3.2.2. MOF-801/Binder Coatings

We then selected two of the binders accelerating the sorption (PVP and bentonite) and prepared MOF-801 coatings with them on the Al foil from MOF-801 powder. During preparation of the coatings, we varied the binder content and the coating thickness. “Thick” coating MOF-801_C/PVP(10)_4.5 was prepared with the *S*/*m* ratio equal to 4.5 m^2^/g, as for the reference monolayer of 0.8–0.9 mm grains of MOF-801. For “thin” coatings, the *S*/*m*-ratio of 8.9 m^2^/g was equal to that for the reference monolayer of 0.4–0.5 mm grains. Similar to loose and glued MOF-801 grains, all kinetics of the water adsorption on and desorption from the coatings (Figure S8 in the Supplementary materials) are well described by Equation (3), with characteristic times *τ*_1_ and *τ*_2_ associated with the initial fast and further slow processes, respectively (Table 3).

**Figure 5:**
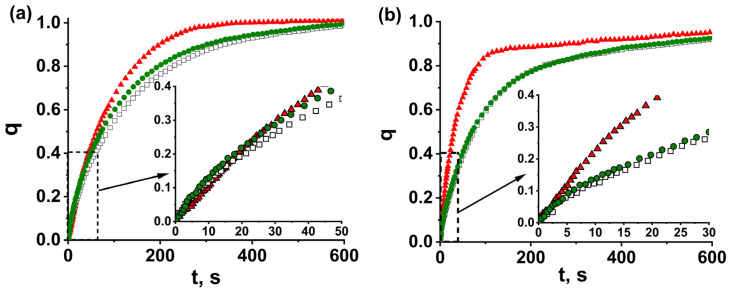
The kinetic curves of water vapor adsorption (**a**) and desorption (**b**) for coatings MOF-801_C/PVP(10)_4.5 (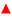), glued grains MOF-801_G/PVP(10)_4.5 (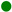) and loose grains MOF-801-G_4.5 (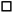).

The comparison of water sorption kinetics for thick beds with *S*/*m* = 4.5 m^2^/kg shows that the ad/desorption for the coating MOF-801_C/PVP(10)_4.5 is faster than for both reference beds of loose grains MOF-801_G_4.5 and glued grains MOF-801_G/PVP_4.5 (Figure 5). The acceleration effect is more significant at longer times (larger conversion), so that the time, *t*_0.8_, decreases by factors of 1.6 and 2.7 for adsorption and desorption, respectively, compared with loose grains (Table 3). The coefficient A = 0.69 for adsorption on the coating is much higher than A = 0.21 and 0.29 for loose and PVP-glued grains, respectively. This indicates that, for coating MOF-801_C/PVP(10)_4.5, the transition from the initial fast process to the further slow one occurs at a higher conversion than for loose grains MOF-801_G_4.5 and glued grains MOF-801_G/PVP_4.5.

**Figure 6:**
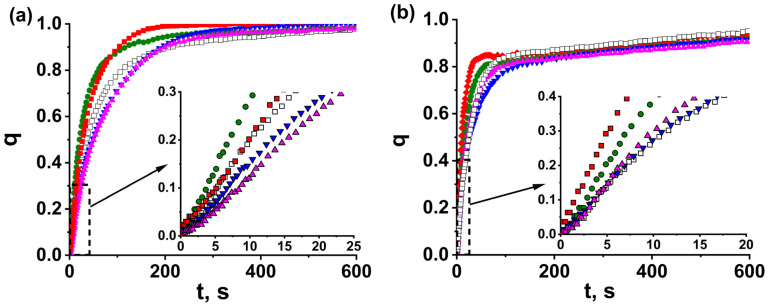
The kinetic curves of water vapor adsorption (**a**) and desorption (**b**) for coatings MOF-801_C/PVP(5)_8.9 (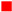), MOF-801_C/PVP(10)_8.9 (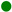), MOF-801_C/bentonite(40)_8.9 (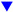), MOF-801_C/bentonite(20)_8.9 (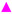), and the reference bed of loose grains MOF-801-G_8.9 (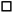).

Unfortunately, we failed to prepare sufficiently strong thick coatings with lower PVP content and bentonite. For this reason, the kinetic data for other coatings were prepared with *S*/*m* = 8.9 and compared with the reference bed MOF-801-G_8.9 of loose grains of 0.4–0.5 mm size (Figure 5). The coatings prepared with PVP (MOF-801_C/PVP(5)_8.9 and MOF-801_C/PVP(10)_8.9) afford essential acceleration of both adsorption and desorption (Figure 5); the characteristic time, *τ*_1_, is reduced by a factor 1.3–1.8 and 1.6–2.2 for adsorption and desorption, respectively (Table 3). A similar trend is observed for times *t*_0.7_ and *t*_0.8_ (Table 3). Surprisingly, the bentonite binder does not accelerate the sorption on the coatings MOF-801_C/bentonite(40)_8.9 and MOF-801_C/bentonite(20)_8.9 compared with the reference bed MOF-801_G_8.9 of loose grains (Figure 6); no reduction of the characteristic times was observed (Table 3).

For both PVP and bentonite, the binder content only slightly affects the sorption kinetics. All “thick” beds ad/desorb water much slower than “thin” beds, which is expected, because they are characterized by a larger ratio, *S*/*m*; consequently, both heat and mass transfer occur faster. The scale of the accelerating effect for the coatings is on a par for thin and thick beds (Figure 5, Table 3).

Let us consider the main processes affecting the sorption kinetics in the system «Al foil–binder–adsorbent». For physical adsorbents, the intrinsic interaction of water molecules with the adsorption site is a very rapid process, and the adsorption kinetics is determined by coupled heat and mass transfer [36]. Since the ad/desorption is initiated by a temperature drop/jump at initial time *t* → 0, the driving temperature difference, Δ*T* = *T*_f_ − *T*_in_, between the heat transfer fluid, *T*_f_, and initial adsorbent temperature, *T*_in_, is maximum. The vapor pressure gradient over the adsorbent is absent; consequently, there is no driving force for mass transfer. Accordingly, at the initial time, the heat transfer controls the sorption rate, and the kinetics is described by time τ_1_. The temperature drop/jump initiates ad/desorption, during which the gradient of water vapor pressure over the adsorbent appears, which is the driving force for the mass transfer. The mass transfer starts to affect the adsorption rate as well, and the transition to the second, slower scenario described by time τ_2_ is detected. For smaller grains MOF-801-G_8.9, the resistance to intraparticle mass transfer is lower than for larger grains MOF-801-G_4.5. Accordingly, the fraction of water adsorbed by the first process, controlled by the heat transfer, is larger (the coefficient A = 0.69 and 0.21 for small and large grains, respectively, Table 2 and Table 3). The desorption runs occur at a higher water vapor pressure *P*_con_ = 43 mbar compared with *P*_ev_ = 9 mbar during adsorption, which contributes to faster mass transfer. As a result, the effect on the desorption rate is detected at a higher conversion, q (larger A values). Gluing the grains with binders only slightly affects the mass transfer in them, and the A values for glued and loose grains are close. On the contrary, for adsorption on coating MOF-801_C/PVP_4.5, the coefficient A = 0.79 is much higher than A = 0.21 and 0.29 for loose grains MOF-801_G_4.5 and glued grains MOF-801_G/PVP_4.5, respectively. A probable reason may be that the coating is made of the as-synthesized microcrystalline powder of MOF-801 with a particle size, D_pp_, of 0.5–1.5 μm, with the particles loosely bonded to each other by PVP. Due to the small amount of PVP, it does not fill the voids between the particles, thus proving enough space for the diffusion of water molecules. On the contrary, pressing the grains results in denser packing of the primary particles, which prevents water diffusion. Accordingly, the resistance to intraparticle mass transfer is smaller for the coating than for larger grains (Figure 1a,c), and its effect on the adsorption kinetics appears at a higher conversion.

### 3.3. Heat Transfer Coefficients

For deeper insight into the processes controlling the adsorption kinetics at initial times, the effective heat transfer coefficient, *U*, is estimated for the studied configurations of the adsorbent bed under conditions of the adsorption cycles. Let us consider the heat balance in the adsorbent bed at *t*→0:

(6) $$U\left(\frac{S}{m_{ad}}\right)\cdot \left(T_{f}-T_{in}\right)=W_{max}+C_{p}\left({dT}_{ad}/dt\right){}_{t=o},$$

where *S* is the surface area of the heat transfer between the adsorbent and the Al foil, *m*_ad_ is the adsorbent mass, *W*_max_ is the specific power consumed/released in the adsorbent bed at *t* → 0, and *C*_pΣ_ is the specific heat capacity of the adsorbent and adsorbed water. Considering that the second summand in Equation (6) is minor, the coefficient *U* can be estimated from the values of *W*_max_, calculated from the initial parts of the kinetic curves:

(7) $$U\left(\frac{S}{m_{ad}}\right)\cdot \left(T_{f}-T_{in}\right)=W_{max}=\Delta H\cdot R_{0}\;=\;{\left.\Delta H\frac{d\Delta w(t)}{dt}\right|}_{t=0}\;=\Delta H\cdot \Delta w_{t\to \propto }/\tau_{1}$$

where Δ*H* is the adsorption heat, equal to 60 kJ/mol [22], and *R*_0_ is the ad/desorption rate at *t* → 0.

**Figure 7:**
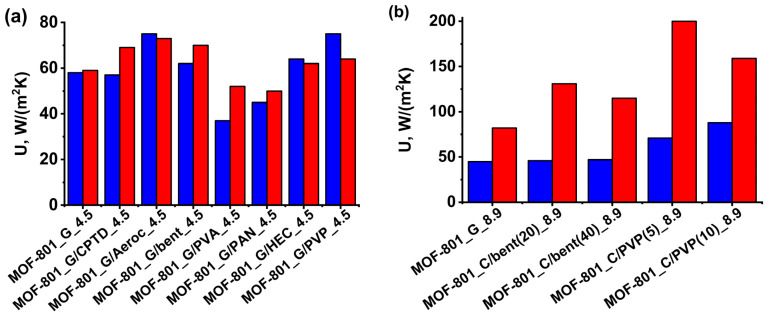
Effective heat transfer coefficients of the water adsorption (blue) and desorption (red) runs for glued grains MOF-801_G/binder_4.5 (**a**) and coatings MOF-801_C/binder_8.9 (**b**).

Gluing the MOF-801 grains using Aerocool, bentonite, HEC, and PVP enhances the effective heat transfer coefficient, *U*, by 7–28% (Figure 7a). The influence of the binder on the effective heat transfer coefficient of thin coatings is even more pronounced. For the PVP-based coatings MOF-801_C/PVP(10)_8.9 and MOF-801_C/PVP(5)_8.9, the coefficient *U* rises by a factor of 1.6–1.9 and 1.9–2.4 for adsorption and desorption, respectively, compared with the reference bed of loose grains MOF-801_G_8.9 (Figure 7b). The enhanced heat transfer results in the speed up of water ad/desorption at the initial time.

Surprisingly, gluing the grains with PVA and PAN does not increase the effective heat transfer coefficient. The overall thermal resistance in the system “Al foil–adsorbent bed” comprises the contact resistances “foil–binder” and “binder–adsorbent”, as well as the resistance of the binder layer. Accordingly, it depends on the adhesion between the foil and binder, between the binder and grains, and on the thermal conductivity of the binder. The thermal conductivity, λ, of inorganic binder bentonite and thermal grease varies in the range 0.83–8.5 W/(mK) [37,38], which is much higher than λ = 0.27–0.32 W/(mK) of organic binders PVA, PVP, PAN, and HEC [39,40]. The high heat conductivity of inorganic binders is expected to contribute to faster heat transfer and adsorption kinetics at initial times. However, the accelerating effect on water adsorption on MOF-801 was observed for binders with both high (bentonite and Aerocool) and low (PVP and HEC) conductivity. Furthermore, the opposite effect of the same binders on the water adsorption dynamics was detected for different adsorbents MOF-801 and LiCl/silica, reported in [41]. Thus, PVP accelerates the water vapor adsorption on MOF-801 and slows down it for LiCl/silica. The CPTD strongly speeds up the sorption on LiCl/silica and has a neutral effect for MOF-801. Probably, the opposite effect of these binders on the effective heat transfer coefficient and the sorption kinetics originates from the adhesion between them and these adsorbents, which strongly depends on the chemical nature of both binder and adsorbent, as well as on the interaction between them. Thus, the choice of the most effective binder is a subject of thorough study for each adsorbent.

### 3.4. Specific Cooling Power

The specific power (SP) is an important performance index of adsorption chillers, which determines the amount of the adsorbent needed and, consequently, the unit volume. To avoid a drop in the power due to sorption deceleration when approaching the equilibrium, in practice, the duration of the working cycle is usually limited by a time corresponding to the conversion q = 0.7–0.8 [9,42]. For the cooling cycle T_ev_/T_con_/T_des_ = 5/30/80 °C restricted by conversion q = 0.7 and 0.8, the SP_q_ is calculated according to the following expression

*SP*_q_ *=* Δ*L*Δ*w*_t→∝_*q*/(*t*_q.ad_
*+ t*_q.des_), (8)

where L is the latent heat of water at *T*_ev_ = 5 °C; and *t*_q.ad_ and *t*_q.des_ are times corresponding to conversion, *q*, for adsorption and desorption, respectively.

The power SP_0.7_ = 1.13 and 2.60 kW/kg can be achieved for the monolayer of loose grains MOF-801_G_4.5 and MOF-801_G_8.9, respectively (Table 4). Gluing the grains to the foil with CPTD, PVA, and PAN does not increase the power. On the contrary, Aerocool, bentonite, HEC, and PVP afford an increase in the SP_0.8_ and SP_0.7_ by 10–20% compared with the reference bed MOF-801_G_4.5 due to the enhancement of the effective heat transfer coefficient, *U* (Figure 6). For the coating MOF-801_C/PVP(10)_4.5, prepared from the suspension, the increase in SP is even larger: 65 and 95% at q = 0.7 and 0.8, respectively. This is in accordance with a larger A value of 0.79, compared with A = 0.29 for MOF-801_G/PVP(10)_4.5 (Table 2 and Table 3), which indicates that the transition to the second slower process occurs at a higher conversion. It can be caused by a lower resistance to the intra-particle mass transfer due to the smaller size of the MOF particles in the coating. The increase in the ratio S/m to 8.9 m^2^/g allows further enhancement of the SCP. For thinner coating MOF-801_C/PVP(5)_8.9, the power SCP = 4.1 and 5.1 can be achieved, which is about twice of that for the reference bed of loose grains MOF-801_G_8.9. The considered configurations may especially be recommended for efficient but expensive adsorbents such as MOFs. In this case, the binder helps to realize fully the sorbent activity and minimize the adsorbent mass and total unit cost.

## 4. Conclusions

The acceleration of the adsorption/desorption stages of adsorption heat transformation and storage (AHTS) cycles and the increase in the specific power (SP) is a key stone for wider distribution of this energy-saving and environmentally friendly technology. This paper is aimed at the acceleration of water vapor adsorption on MOF-801, a promising adsorbent for adsorption cooling, the optimization of the bed configuration, and of SP enhancement. The dynamics of water vapor adsorption on MOF-801 under conditions of adsorption/desorption stages of a typical cooling cycle is studied and compared for three adsorbent bed configurations, namely, the monolayer of loose MOF-801 grains located on an aluminum support, the monolayer of grains glued to the support, and thin coatings prepared on the support surface with binders.

It is shown that, for all studied configurations, the ad/desorption kinetics are described by the sum of two exponents with rather different characteristic times, which indicates a change in the rate-limiting stage during the process. The heat transfer between the adsorbent and aluminum support controls the adsorption rate at the initial time. At longer times, the mass transfer in the adsorbent grains/coatings starts to affect the sorption kinetics. Gluing the grains using PVP, HEC, Aerocool, and bentonite affords an increase in the effective heat transfer coefficient between the support and MOF-801 of 7–28%. For PVP-based thin coatings, even stronger growth of the heat transfer coefficient by a factor of 1.6–2.4 is observed compared with loose grains. The enhanced heat transfer results in the speed up of the water ad/desorption and the growth of the SP_q_, achievable in the adsorption cycle, restricted by conversion *q* = 0.7 and 0.8. Gluing the grains with HEC, PVP, Aerocool, and bentonite affords an increase in the SP_q_ of 10–20%. For the thin coatings prepared with PVP, an SP_q_ of 1.64–5.16 kW/kg can be achieved, which is about twice as high as that for the reference beds of loose grains.

## Figures and Tables

**Table 1:** Composition and texture characteristics of the prepared MOF-801 beds. Binder content, *C*_b_, specific surface area, *S*_sp_, total pore volume, *V*_p_, micropore volume, *V*_μ_, and clogging degree, δ.

Sample	*C*_b_ [wt%]	*S*_sp_ * [m^2^/g]	*V*_p_ * [cm^3^/g]	*V*_μ_ * [cm^3^/g]	δ, %
MOF-801 as prepared	-	900	0.49	0.27	-
MOF-801_G	-	820	0.45	0.23	-
Glued grains, 0.8–0.9 mm					
MOF-801_G/PVA	10	757	0.43	0.22	4
MOF-801_G/PVP	10	774	0.45	0.24	0
MOF-801_G/PAN	5	803	0.46	0.24	0
MOF-801_G/AON	40	724	0.41	0.21	9
MOF-801_G/bent	40	863	0.57	0.24	−27
MOF-801_G/CPTD	40	380	0.32	0.11	29
MOF-801_G/Aerocool	40	732	0.42	0.27	7
MOF-801_G/HEC	10	695	0.40	0.19	11
Coatings					
MOF-801_C/PVP(5)	5	858	0.44	0.26	10
MOF-801_C/PVP(10)	10	830	0.43	0.25	12
MOF-801_C/bent(20)	20	954	0.54	0.28	−10
MOF-801_C/bent(40)	40	1089	0.67	0.37	−37

* For consolidated layers prepared with binders, the data are related to the unit mass of the MOF.

**Table 2:** The characteristic times of water adsorption/desorption τ_1_, τ_2_, τ_0.7_ and τ_0.8_ and the final water uptake, Δ*w*_t→∝_, for the glued grains of MOF-801, D_gr_ = 0.8–0.9 mm.

Configuration	*τ*_1_, s	*τ*_2_, s	A	*t*_0.7_, s	*t*_0.8_, s	Δ*w*_t→∝_, * g/g
Adsorption						
MOF-801_G_4.5	88	170	0.21	170	245	0.21
MOF-801_G/CPTD_4.5	87	210	0.29	185	270	0.20
MOF-801_G/Aerocool_4.5	67	160	0.23	140	210	0.20
MOF-801_G/bent_4.5	81	150	0.15	155	220	0.22
MOF-801_G/PVA_4.5	130	330	0.21	320	460	0.20
MOF-801_G/PVP_4.5	69	160	0.29	140	200	0.21
MOF-801_G/PAN_4.5	113	200	0.15	220	300	0.20
MOF-801_G/HEC_4.5	77	130	0.20	130	185	0.20
Desorption						
MOF-801-G_4.5	117	480	0.68	150	240	0.23
MOF-801_G/CPTD_4.5	98	290	0.61	130	200	0.23
MOF-801_G/Aerocool_4.5	91	310	0.64	115	185	0.22
MOF-801_G/bent_4.5	103	300	0.63	130	195	0.24
MOF-801_G/PVA_4.5	127	320	0.69	150	215	0.22
MOF-801_G/PVP_4.5	108	460	0.70	145	235	0.23
MOF-801_G/PAN_4.5	145	270	0.53	175	250	0.24
MOF-801_G/HEC_4.5	106	360	0.67	135	205	0.22

* The uptake is related to the unit mass of MOF-801.

**Table 3:** The characteristic times of water adsorption/desorption τ_1_, τ_2_, τ_0.7_, and τ_0.8_ and the final water uptake, Δ*w*_t→∝_, for MOF-801 coatings.

Sample	*τ*_1_, s	*τ*_2_, s	A	*t*_0.7_, s	*t*_0.8_, s	Δ*w*_t→∝_, * g/g
Adsorption						
MOF-801-G_4.5	88	173	0.21	170	245	0.21
MOF-801_C/PVP(10)_ 4.5	87	241	0.79	115	148	0.20
MOF-801-G_8.9	50	155	0.61	72	111	0.18
MOF-801_C/PVP(10)_8.9	28	68	0.60	40	59	0.20
MOF-801_C/PVP(5)_8.9	38	49	0.18	48	67	0.22
MOF-801_C/bent(40)_8.9	58	94	0.18	94	134	0.22
MOF-801_C/bent(20)_8.9	59	84	0.15	98	135	0.22
Desorption						
MOF-801-G_4.5	103	476	0.68	150	240	0.23
MOF-801_C/PVP(10)_4.5	50	540	0.82	61	88	0.21
MOF-801-G_8.9	33	507	0.78	47	69	0.18
MOF-801_C/PVP(10)_8.9	20	602	0.77	32	56	0.21
MOF-801_C/PVP(5)_8.9	15	637	0.80	21	31	0.23
MOF-801_C/bent(40)_8.9	31	633	0.76	64	122	0.24
MOF-801_C/bent(20)_8.9	27	787	0.77	49	94	0.24

* The uptake is related to the unit mass of MOF-801.

**Table 4:** The specific powers W_max_, SCP_0.8_, and SCP_0.7_ for the cooling cycles *T*_ev_/*T*_con_/*T*_des_ = 5/30/80 °C, respectively.

Configuration	W_max_, kW/kg		SCP_0.8_, kW/kg	SCP_0.7_, kW/kg
	Adsorption	Desorption		
Loose grains				
MOF-801_G_4.5	7.8	7.4	0.85	1.13
MOF-801_G_8.9	12.0	18.3	1.95	2.60
Glued grains				
MOF-801_G/CPTD_4.5	7.6	7.7	0.84	1.10
MOF-801_G/Aerocool_4.5	10.0	8.1	1.01	1.35
MOF-801_G/PVA_4.5	5.0	5.8	0.58	0.72
MOF-801_G/bentonite_4.5	8.3	7.7	1.06	1.31
MOF-801_G/HEC_4.5	8.6	6.9	1.01	1.30
MOF-801_G/PAN_4.5	5.9	5.5	0.73	0.90
MOF-801_G/PVP_4.5	10.0	7.1	0.95	1.24
Coatings				
MOF-801_C/PVP(10)_4.5	7.9	13.8	1.66	1.99
MOF-801_C/PVP(10)_8.9	23.6	35.4	3.26	4.60
MOF-801_C/PVP(5)_8.9	18.8	44.6	4.09	5.16
MOF-801_C/bent(40)_8.9	12.7	25.7	1.64	2.32
MOF-801_C/bent(20)_8.9	12.3	29.2	1.86	2.54

## Data Availability

The data presented in this study are available on request from the corresponding author.

## Supplementary Materials

The following supporting information can be downloaded at: https://www.mdpi.com/article/10.3390/nano13172442/s1, Figure S1: Characteristic curve of water adsorption (a) and isosters (b) of water vapour adsorption on as-prepared MOF-801 powder. Dashed blue lines (b) represent the adsorption cooling cycle, Figure S2: Schematic presentation of the preparation of MOF-801 beds composed of glued grains and coatings, Figure S3: The kinetic curves of water vapour adsorption for the loose grains placed on the surface of aluminum foil and placed on the bottom of the measuring cell, Figure S4: Isotherms of N2 adsorption on as-prepared MOF-801 and pressed grains (a). Experimental and calculated from single-crystal X-ray data (CIF-file) PXRD patterns of the synthesized MOF-801 (b), Figure S5: Pore size distribution of as-prepared MOF-801 powder (a), MOF-801_G (b), MOF-801_G/AON_4.5 (c), MOF-801_G/bentonite_4.5 (d), MOF-801_G/HEC_4.5 (e), MOF-801_G/CPTD_4.5 (f), MOF-801_G/PAN_4.5 (g), and MOF-801_G/PVA_4.5 (h), Figure S6: The kinetic curves of water adsorption on (a,c) and desorption from (b,d) reference beds of loose grains MOF-801_G_4.5 (a,b) and MOF-801_G_8.9 (c,d), Figure S7: The kinetic curves of water adsorption on (left) and desorption from (right) beds of glued grains MOF-801_G/PVP_4.5 (a,b), MOF-801_G/bentonite_4.5 (c,d), MOF-801_G/CPTD_4.5 (e,f), MOF-801_G/PAN_4.5 (g,h), MOF-801_G/PVA_4.5 (i,j), MOF-801_G/Aerocool_4.5 (k,l), and MOF-801_G/HEC_4.5 (m,n).
