# Supplementary material for: Water Adsorption Dynamics on Metal–Organic Framework MOF-801: Comparative Study of Loose and Glued Grains, and Coatings
Source: Nanomaterials (Basel). 2023 Aug 28;13(17):2442. doi: 10.3390/nano13172442 (PMC10490435; doi:10.3390/nano13172442)
Supplement: Supplementary file 1 [file nanomaterials-13-02442-s001.zip › nanomaterials-2569168-supplementary.pdf]

Supplementary Materials

Water adsorption dynamics on metal-organic framework MOF-801: comparative study of loose, glued grains, and coatings

Marina V. Solovyeva*, Larisa G. Gordeeva

Boreskov Institute of Catalysis, Ak. Lavrentiev av. 5, Novosibirsk, Russia

*Corresponding author, e-mail address: solovyeva@catalysis.ru

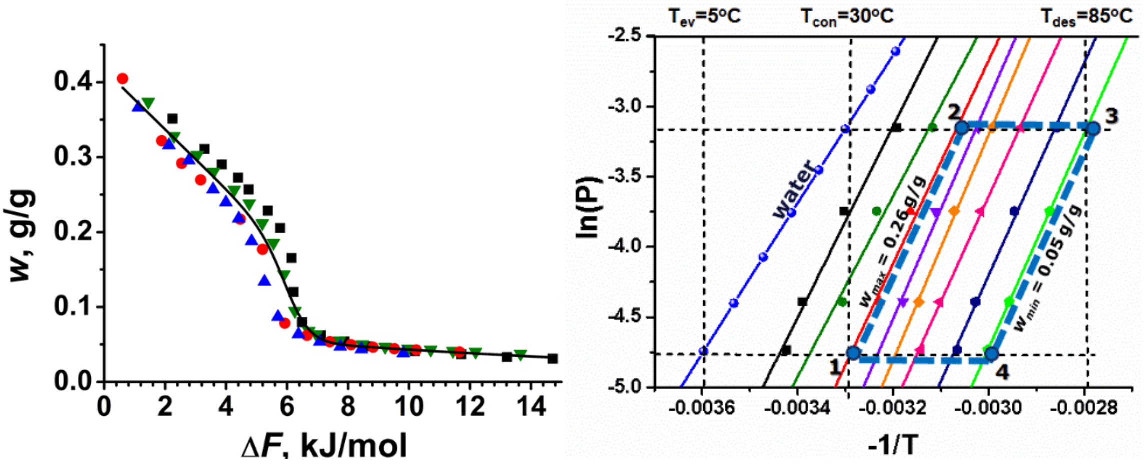

(a) (b)

Figure S1. Characteristic curve of water adsorption (a) and isosters (b) of water vapour adsorption on as-prepared MOF-801 powder. Dashed blue lines (b) represent the adsorption cooling cycle. Reprinted from Energy Conv. Manag., vol. 174, Solovyeva M., Gordeeva L., MOF-801 as a promising material for adsorption cooling: Equilibrium and dynamics of water adsorption, pages 356-363, copyright 2018, with permission of Elsevier.

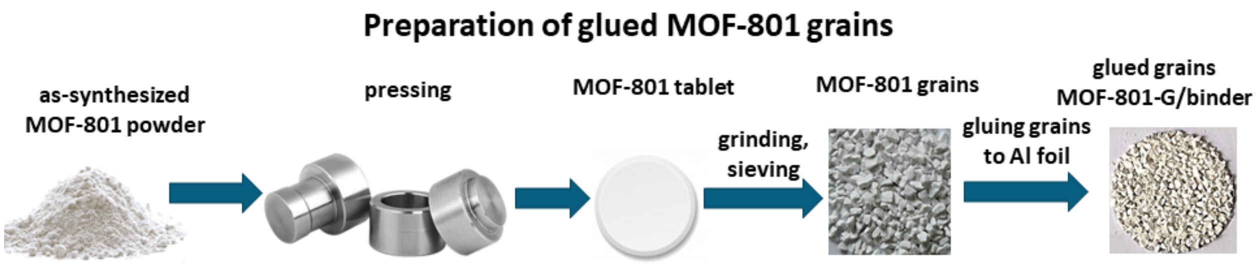

Figure S2. Schematic presentation of the preparation of MOF-801 beds composed of glued grains and coatings.

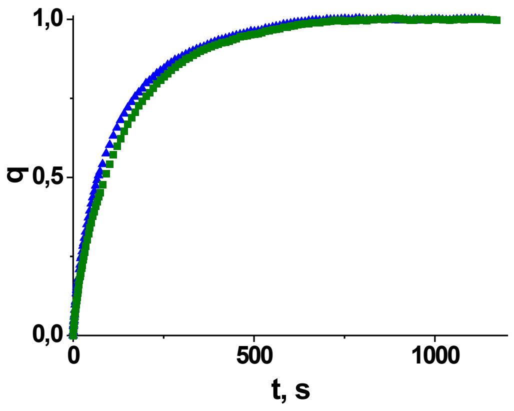

Figure S3. The kinetic curves of water vapour adsorption for the loose grains placed on the surface of aluminum foil (■) and placed on the bottom of the measuring cell (▲).

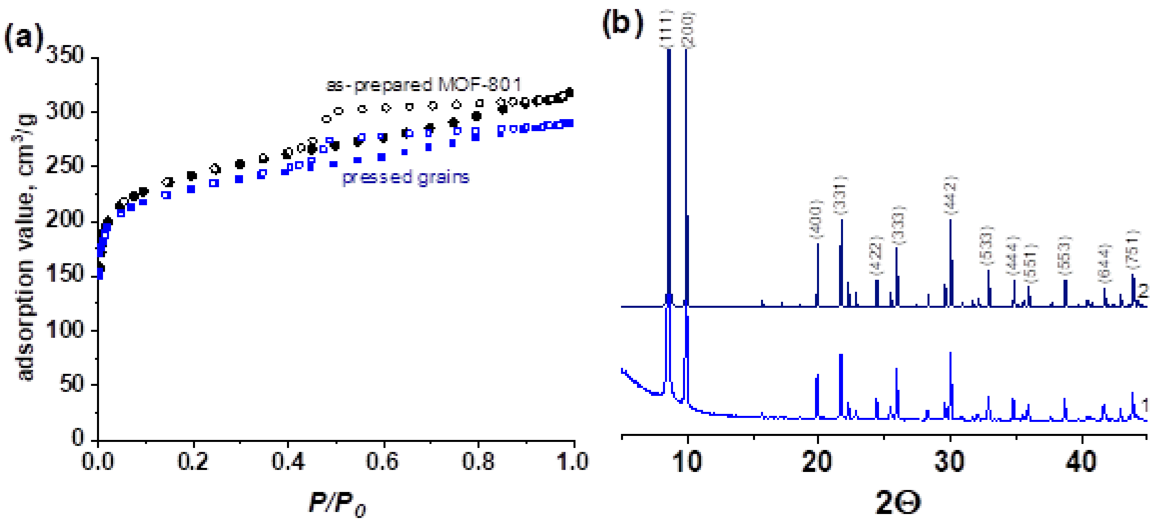

Figure S4. Isotherms of N_2 adsorption on as-prepared MOF-801 and pressed grains (a). Experimental (1) and calculated from single-crystal X-ray data (CIF-file) (2) PXRD patterns of the synthesized MOF-801 (b).

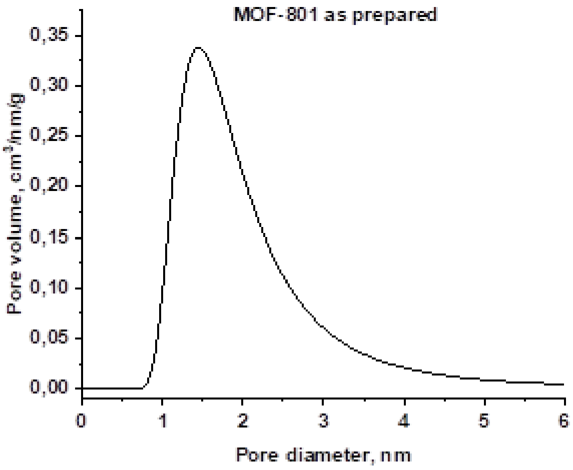

(a)

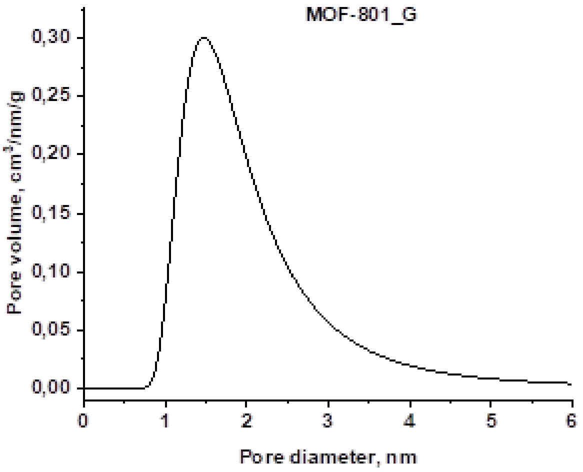

(b)

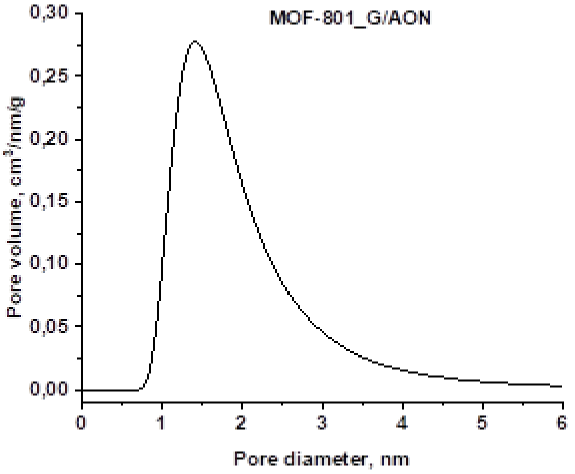

(c)

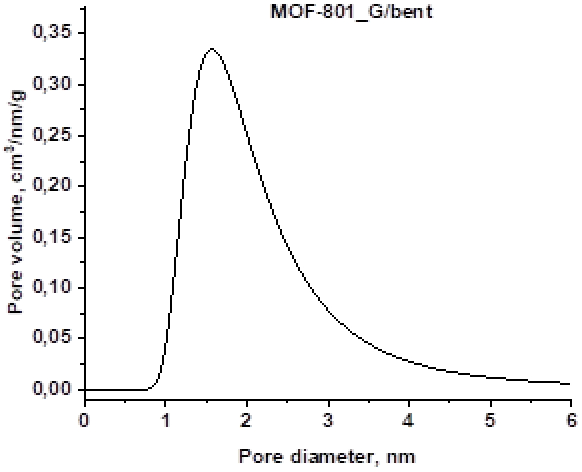

(d)

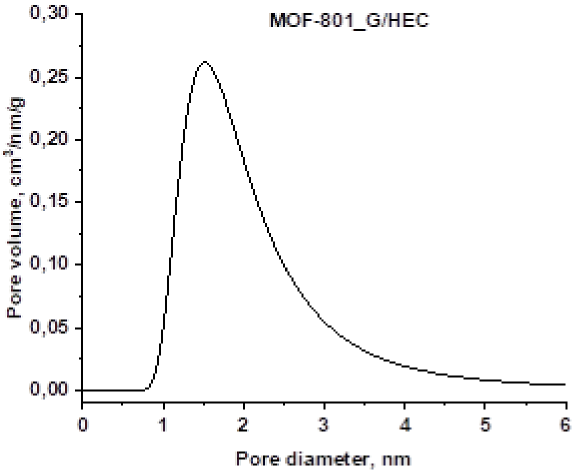

(e)

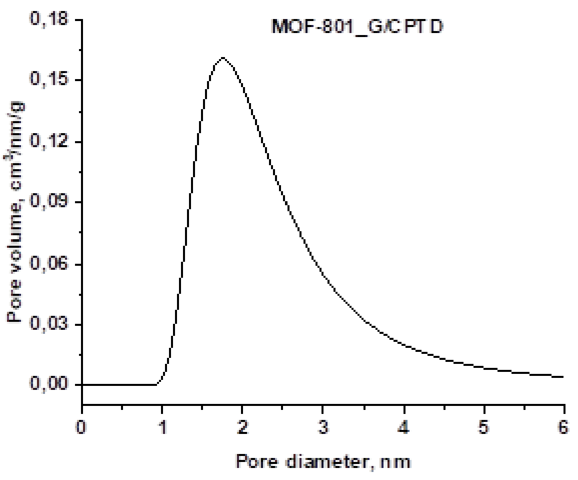

(f)

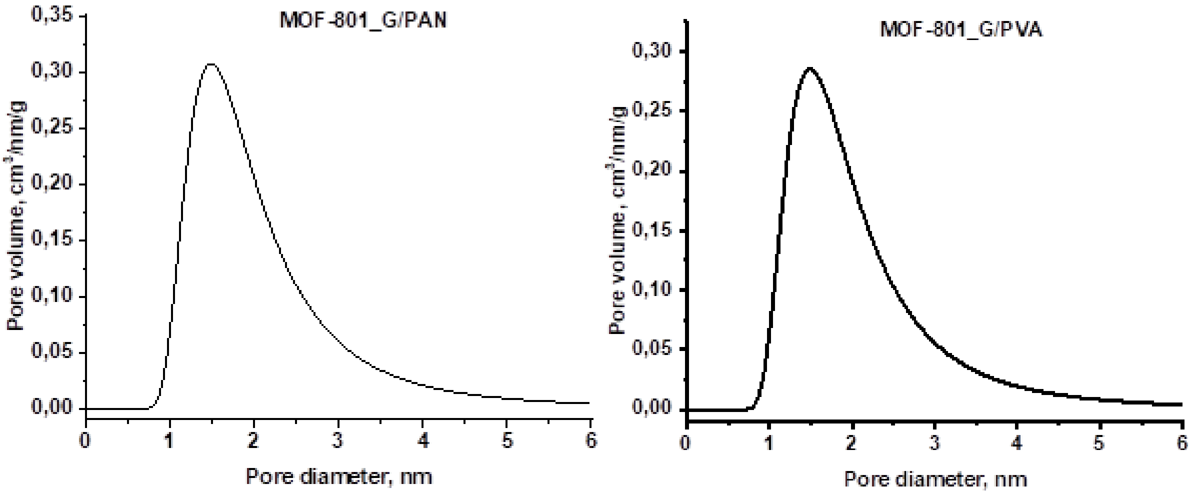

(g) (h)

Figure S5. Pore size distribution of as-prepared MOF-801 powder (a), MOF-801_G (b), MOF-801_G/AON_4.5 (c), MOF-801_G/bentonite_4.5 (d), MOF-801_G/HEC_4.5 (e), MOF-801_G/CPTD_4.5 (f), MOF-801_G/PAN_4.5 (g), and MOF-801_G/PVA_4.5 (h).

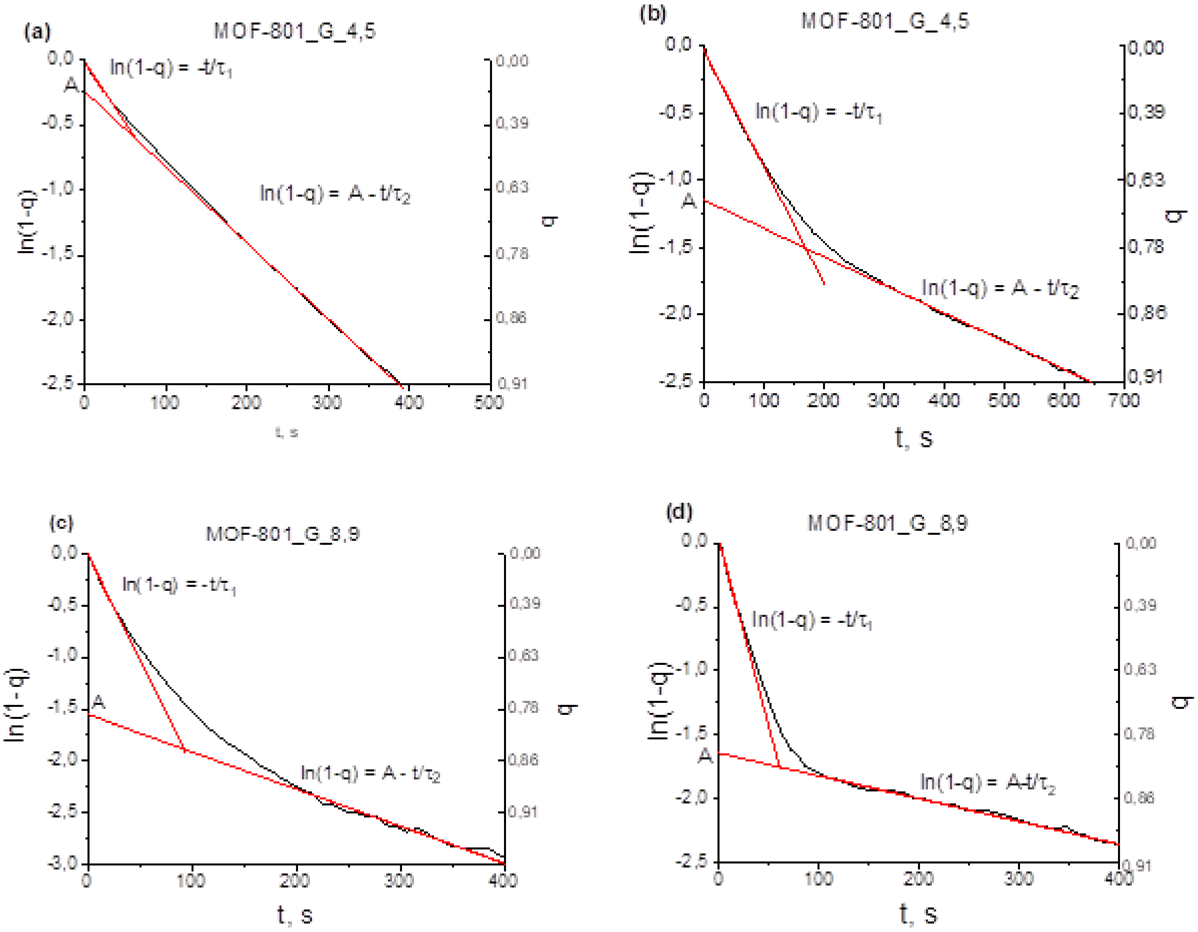

Figure S6. The kinetic curves of water adsorption on (a, c) and desorption from (b, d) reference beds of loose grains MOF-801_G_4.5 (a, b) and MOF-801_G_8.9 (c, d).

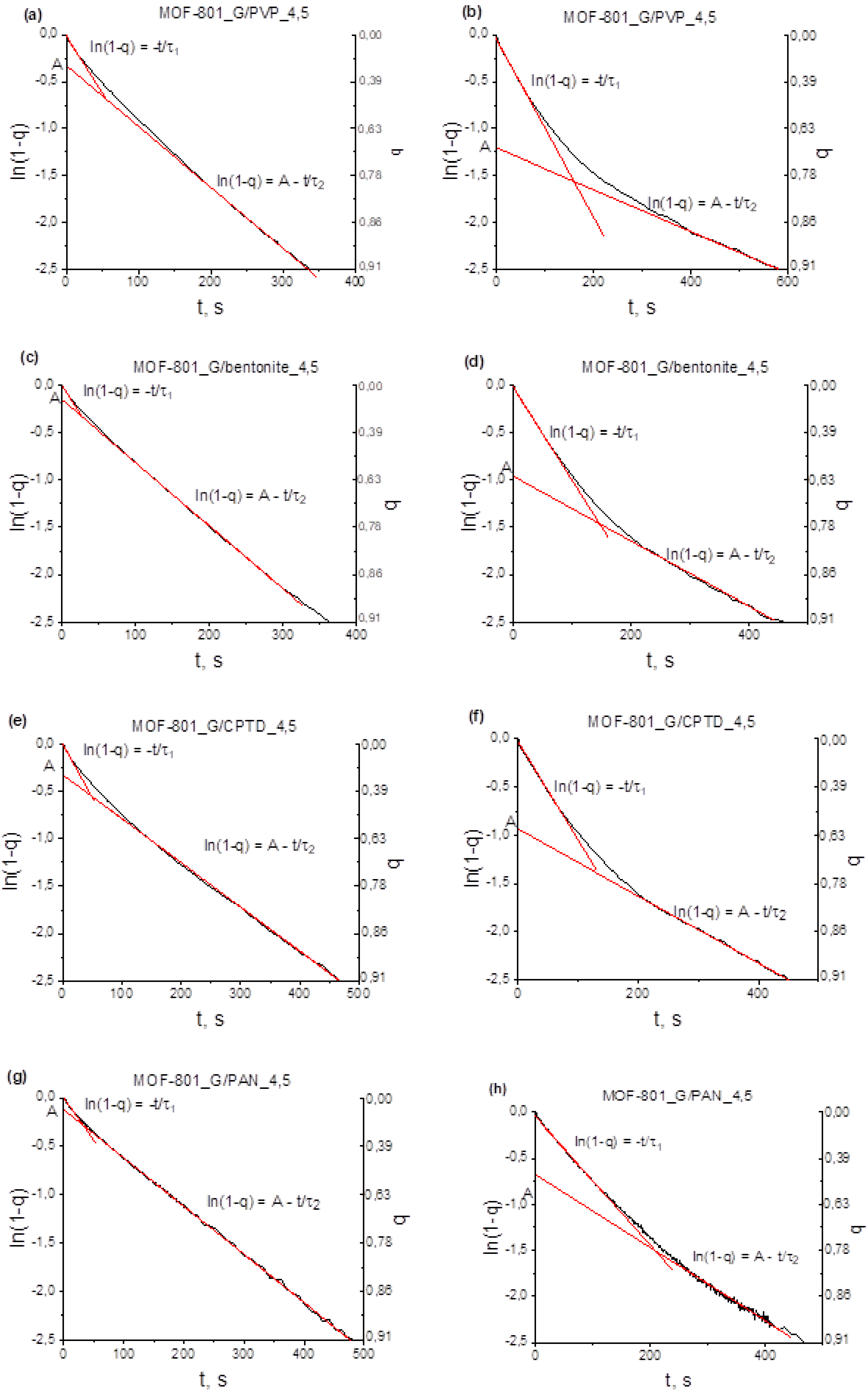
(a)
MOF-801_G/PVP_4,5
ln(1-q) = -t/τ1
ln(1-q) = A - t/τ2
A
ln(1-q)
q
t, s
(b)
MOF-801_G/PVP_4,5
ln(1-q) = -t/τ1
ln(1-q) = A - t/τ2
A
ln(1-q)
q
t, s
(c)
MOF-801_G/bentonite_4,5
ln(1-q) = -t/τ1
ln(1-q) = A - t/τ2
A
ln(1-q)
q
t, s
(d)
MOF-801_G/bentonite_4,5
ln(1-q) = -t/τ1
ln(1-q) = A - t/τ2
A
ln(1-q)
q
t, s
(e)
MOF-801_G/CPTD_4,5
ln(1-q) = -t/τ1
ln(1-q) = A - t/τ2
A
ln(1-q)
q
t, s
(f)
MOF-801_G/CPTD_4,5
ln(1-q) = -t/τ1
ln(1-q) = A - t/τ2
A
ln(1-q)
q
t, s
(g)
MOF-801_G/PAN_4,5
ln(1-q) = -t/τ1
ln(1-q) = A - t/τ2
A
ln(1-q)
q
t, s
(h)
MOF-801_G/PAN_4,5
ln(1-q) = -t/τ1
ln(1-q) = A - t/τ2
A
ln(1-q)
q
t, s

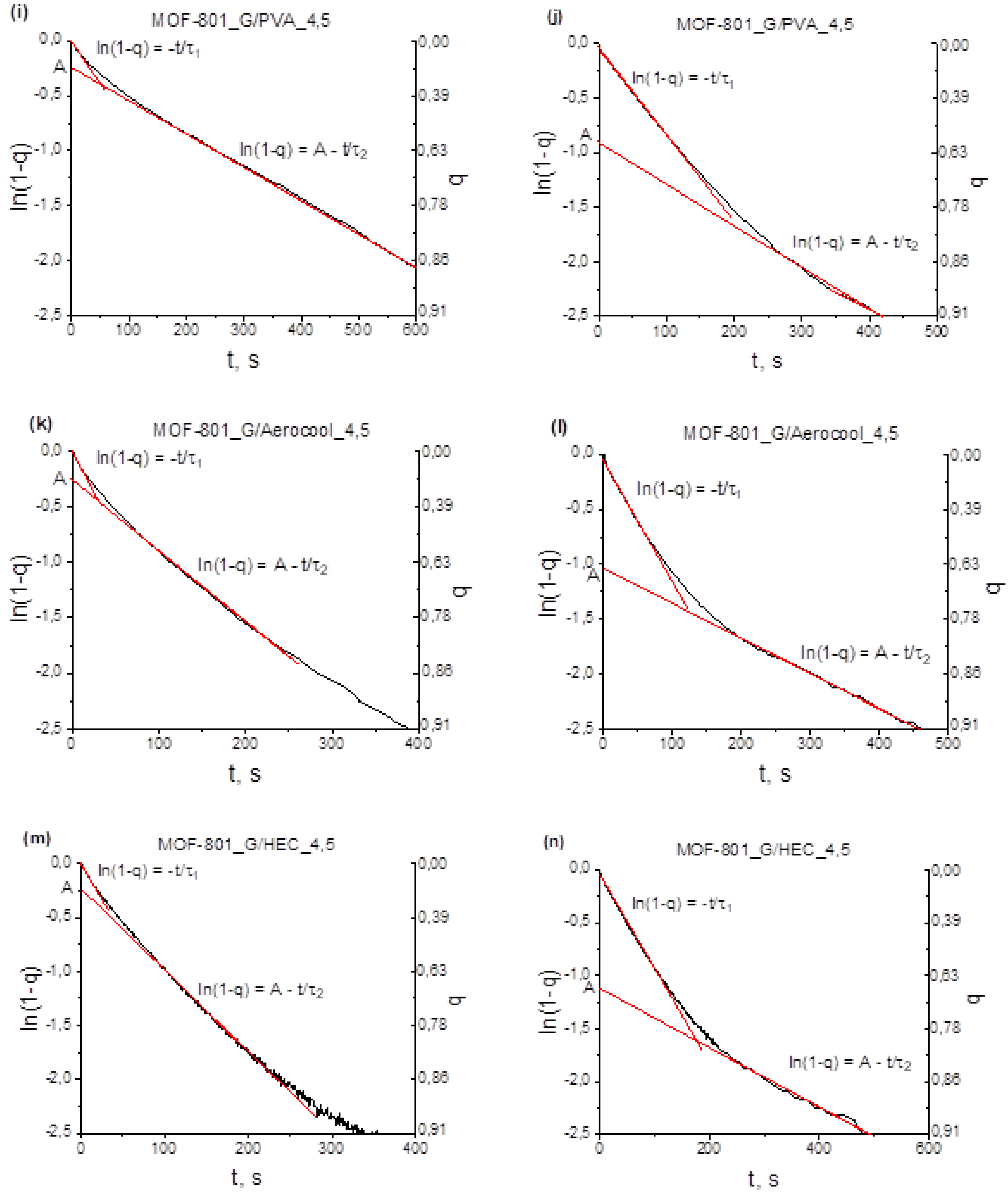

Figure S7. The kinetic curves of water adsorption on (left) and desorption from (right) beds of glued grains MOF-801_G/PVP_4.5 (a, b), MOF-801_G/bentonite_4.5 (c, d), MOF-801_G/CPTD_4.5 (e, f), MOF-801_G/PAN_4.5 (g, h), MOF-801_G/PVA_4.5 (i, j), MOF-801_G/Aerocool_4.5 (k, l), and MOF-801_G/HEC_4.5 (m, n).

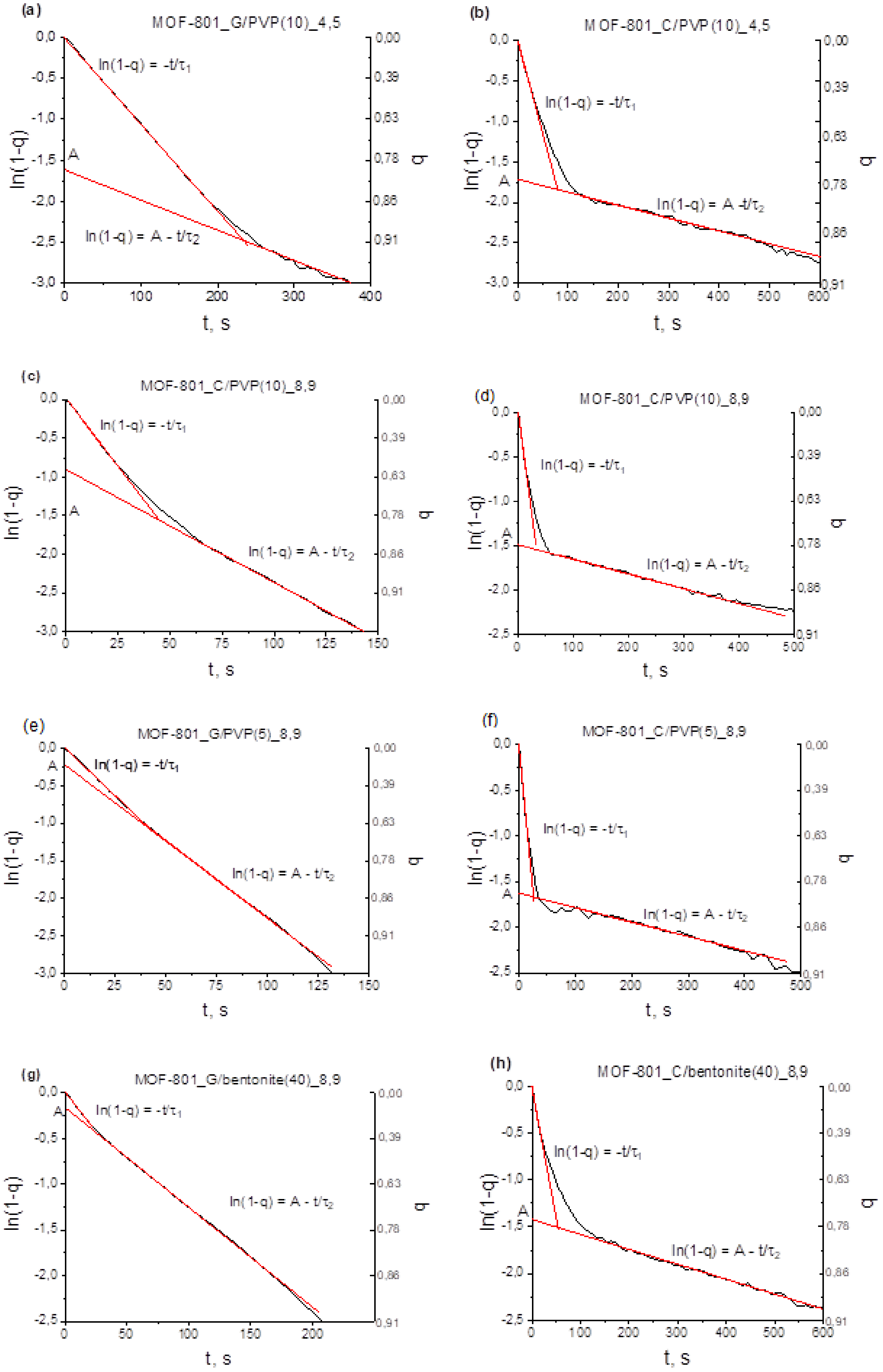

(a)
MOF-801_G/PVP(10)_4,5
ln(1-q) = -t/τ1
ln(1-q) = A - t/τ2
A
ln(1-q)
q
t, s
(b)
MOF-801_C/PVP(10)_4,5
ln(1-q) = -t/τ1
ln(1-q) = A -t/τ2
A
ln(1-q)
q
t, s
(c)
MOF-801_C/PVP(10)_8,9
ln(1-q) = -t/τ1
ln(1-q) = A - t/τ2
A
ln(1-q)
q
t, s
(d)
MOF-801_C/PVP(10)_8,9
ln(1-q) = -t/τ1
ln(1-q) = A - t/τ2
A
ln(1-q)
q
t, s
(e)
MOF-801_G/PVP(5)_8,9
ln(1-q) = -t/τ1
ln(1-q) = A - t/τ2
A
ln(1-q)
q
t, s
(f)
MOF-801_C/PVP(5)_8,9
ln(1-q) = -t/τ1
ln(1-q) = A - t/τ2
A
ln(1-q)
q
t, s
(g)
MOF-801_G/bentonite(40)_8,9
ln(1-q) = -t/τ1
ln(1-q) = A - t/τ2
A
ln(1-q)
q
t, s
(h)
MOF-801_C/bentonite(40)_8,9
ln(1-q) = -t/τ1
ln(1-q) = A - t/τ2
A
ln(1-q)
q
t, s

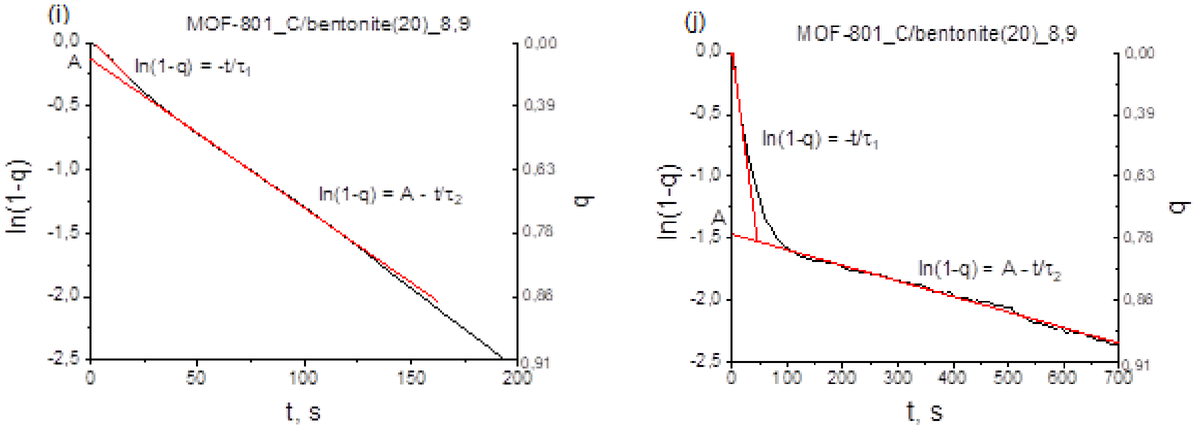

Figure S8. The kinetic curves of water adsorption on (left) and desorption from (right) beds of coatings MOF-801_C/PVP(10)_4.5 (a, b), MOF-801_C/PVP(10)_8.9 (c, d), MOF-801_C/PVP(5)_8.9 (e, f), MOF-801_C/bentonite(40)_8.9 (g, h), and MOF-801_C/bentonite(20)_8.9 (i, j).
